# Genome Sequences of Four *Staphylococcus aureus* Strains Isolated from Bovine Mastitis

**DOI:** 10.1128/genomeA.00334-15

**Published:** 2015-04-23

**Authors:** Ravi Kant, Suvi Taponen, Joanna Koort, Lars Paulin, Silja Åvall-Jääskeläinen, Airi Palva

**Affiliations:** a Department of Veterinary Biosciences, Faculty of Veterinary Medicine, University of Helsinki, Helsinki, Finland; b Department of Production Animal Medicine, Faculty of Veterinary Medicine, University of Helsinki, Helsinki, Finland; c Institute of Biotechnology, University of Helsinki, Helsinki, Finland

## Abstract

*Staphylococcus aureus* is a major causative agent of mastitis in dairy cows. The pathogenicity of *S. aureus* may vary; it is able to cause severe clinical mastitis, but most often it is associated with chronic subclinical mastitis. Here, we present the genome assemblies of four *S. aureus* strains from bovine mastitis.

## GENOME ANNOUNCEMENT

*Staphylococcus aureus* is part of skin and mucosal microbiota but also an opportunistic pathogen causing disease both in humans and different animal species (1). In bovines, it causes mastitis with symptoms that may vary from subclinical to severe clinical. Typically, *S. aureus* mastitis causes a moderate to strong increase in milk somatic cell count and thus impairs milk quality (2). In Finland and worldwide, *S. aureus* is the most common cause of clinical mastitis, and the second most common bacterium isolated and identified from subclinical mastitis, after coagulase-negative staphylococci (2, 3).

*S. aureus* strains 9, 75, 110, and 112 used in this study were isolated from mastitic milk samples of four cows on four commercial dairy herds during veterinary practice at the Ambulatory Clinic of the Faculty of Veterinary Medicine, University of Helsinki, Finland. Two strains (9 and 75) originated from moderate to severe clinical mastitis and two (110 and 112) from subclinical mastitis. The genomic DNA was extracted using the Easy-DNA kit for genomic DNA isolation (Invitrogen Life Technologies, Carlsbad, CA, USA), and the entire genomes were sequenced using the Roche 454 Life Sciences GS FLX system. The obtained sequences were assembled using Newbler, resulting in 18×, 23×, 28×, and 20× coverage of each genome, respectively.

Coding sequences (CDSs) were predicted using a combination of GeneMark and Glimmer3 (4, 5) followed by manual curation of the start sites of open reading frames. The remaining intergenic regions were reanalyzed for missed CDSs by using BLASTx (6). Annotation transfer was performed based on the BLASTp search, followed by RAST (Rapid Annotations using Subsystem Technology) analysis (7) and manual verification.

The draft genomes of *S. aureus* strains 9, 75, 110, and 112 contain 2,699,547, 2,650,629, 2,660,305, and 2,646,563 nucleotides, respectively. The overall G+C content of all four genomes is 32.6%. The genomes contain 2,478, 2,544, 2,547, and 2,469 protein-coding sequences (CDS) with 59, 56, 62, and 66 RNAs, respectively. All these values are quite similar with the corresponding values in the genome statistics of *S. aureus* compiled by the Broad Institute (http://www.broadinstitute.org/annotation/genome/staphylococcus_aureus_group/GenomeStats.html). The pan-genome consists of only 2,815 genes and the core genome of 2,254 genes. However, when compared with other publically available *S. aureus* genomes, we identified a pan-genome of 5,489 genes and a core genome of 1,430 genes.

Putative functions were predicted for 1,966 (79%), 2,040 (80%), 2,046 (80%), and 1,993 (81%) CDSs of the strains 9, 75, 110, and 112, while 512 (21%), 504 (20%), 501 (20%), and 476 (19%) CDSs of these strains were found to be hypothetical or of unknown function, respectively. Using the SignalP software (8), a low percentage of CDSs (about 6.5%) of our four *S. aureus* strains were predicted to be secreted (161, 160, 166, and 160, respectively) and thus belong to genes encoding proteins involved in, for example, host-microbe interactions.

### Nucleotide sequence accession numbers. 

The draft genome sequences of *Staphylococcus aureus* 9, *Staphylococcus aureus* 75, *Staphylococcus aureus* 110, and *Staphylococcus aureus* 112 are available in GenBank under the accession numbers JZIN00000000, JZIO00000000, JZIP00000000, and JZIQ00000000, respectively.
